# A systematic review of alterations in sensorimotor networks following stroke: implications for integration and functional outcomes across recovery stages

**DOI:** 10.3389/fneur.2025.1456146

**Published:** 2025-05-27

**Authors:** Nur Shaheera Aidilla Sahrizan, Noorazrul Yahya, Zhe Kang Law, Wan Asyraf Wan Zaidi, Umi Nabilah Ismail, Naela Himayati Afifah, Amirul Azri, Hanani Abdul Manan

**Affiliations:** ^1^Makmal Pemprosesan Imej Kefungsian (Functional Image Processing Laboratory), Department of Radiology, Universiti Kebangsaan Malaysia, Kuala Lumpur, Malaysia; ^2^Diagnostic Imaging and Radiotherapy Program, School of Diagnostic and Applied Health Sciences, Faculty of Health Sciences, Universiti Kebangsaan Malaysia, Kuala Lumpur, Malaysia; ^3^Neurology Unit, Department of Medicine, Faculty of Medicine, Universiti Kebangsaan Malaysia, Kuala Lumpur, Malaysia; ^4^Department of Radiology and Intervency, Hospital Pakar Kanak-Kanak (Children Specialist Hospital), Universiti Kebangsaan Malaysia, Kuala Lumpur, Malaysia

**Keywords:** stroke, functional connectivity, sensorimotor networks, Fugl-Mayer Assessment, functional magnetic resonance imaging

## Abstract

**Introduction:**

Stroke can result in a wide range of impairments, with sensorimotor dysfunction being among the most common, particularly when the sensorimotor network (SMN) is affected. As the SMN plays a critical role in movement control and coordination, understanding the changes in this network post-stroke is essential for informing recovery and rehabilitation strategies.

**Methods:**

A systematic review was conducted following PRISMA guidelines. Two electronic databases, PubMed and Scopus, were searched for relevant studies investigating the effects of stroke on the SMN across different phases of recovery. Reference lists of selected articles were also reviewed using Google Scholar. A total of 20 eligible studies involving 618 stroke patients and 606 healthy controls were included.

**Results:**

The review revealed consistent findings of altered functional connectivity within the SMN following stroke. Despite initial impairments, most studies reported improvement in SMN connectivity over time, attributed to compensatory mechanisms, cortical reorganisation, and functional rewiring. Stroke location significantly influenced recovery outcomes. Supratentorial strokes were associated with poorer motor assessments and slower recovery, while infratentorial strokes had comparatively better outcomes. Lesions in the pontine region were found to cause severe disturbances in both sensory and motor functions depending on lesion extent.

**Discussion:**

The findings underscore the brain's capacity for neuroplasticity and reorganisation following stroke. Understanding the temporal and spatial changes in the SMN post-stroke can inform more targeted and effective rehabilitation strategies. These insights are crucial for tailoring interventions that align with individual stroke profiles and promote optimal functional recovery.

## Highlights

- The motor cortex, integral to the sensorimotor network (SMN) is directly affected by stroke, contributing to muscle weakness and challenges in executing specific motor tasks.
- The correlation between motor deficiencies and altered brain activity across multiple neural networks showed potential dysfunctions in complex motor behaviors even in well-recovered patients.
- Disruptions in connections within the SMN contribute to a global alteration in the functioning of this neural network.
- Activation patterns in the contralateral and ipsilateral SMC and premotor cortex may signify variances in cortical reorganization during the chronic phase.

## 1 Introduction

A stroke is a medical emergency caused by disruption of blood supply due to blood vessel occlusion or bleeding due to burst blood vessels, resulting in the death of brain cells. After a stroke, changes in the brain's neuronal network connectivity have been observed (1). Studies have shown that the brain network may evolve to a more complex and random mode during the process of rehabilitation after a stroke (1–3). This evolution involves the production of new connections to compensate for damaged connections and nerves, as well as the reorganization of functional network connectivity, which is central to the process of rehabilitation. The sensorimotor network (SMN) plays a crucial role in daily functioning, encompassing the integration of sensory and motor formation to execute purposeful movements. These networks include the primary motor cortex (M1), the somatosensory cortex, the premotor cortex (PMC), and the supplementary motor area (SMA).

The SMN is responsible for the planning, execution, and control of voluntary movements, and the somatosensation (4). The SMN is also involved in the processing of proprioceptive information, which is essential for the perception of body position and movement (5). The SMN is connected to wider functional networks that coordinate the activity of separate cortical regions. These pathways link the SMN to other brain regions involved in higher-order cognitive processes, such as attention, memory, and decision-making (6, 7) The SMN is also connected to subcortical structures, including the basal ganglia and the cerebellum, which is pivotal in motor control and learning (8). Therefore, it is important to evaluate the post-stroke changes and the recovery mechanism particularly strokes that involve this SMN.

Research has shown that the impact of stroke depends on the location of the stroke (9, 10). Stroke can lead to sensorimotor deficits, affecting the ability to incorporate sensory inputs for motor output, thereby compromising goal-directed functional movements and motor abilities (11–13). The significance of SMN in daily functioning is evident in the context of stroke, as they are essential for activities of daily living and overall quality of life. Stroke survivors often experience motor and sensory impairments, cognitive deficits, and emotional disorders, which can significantly impact their independence and wellbeing (14). Stroke-induced damage to the M1 can result in weakness or paralysis of the contralateral limbs. However, the brain can undergo reorganization and plasticity to compensate for the damage, leading to the recruitment of alternative motor pathways and the development of new connections between brain regions (15). The recovery process after stroke involves multiple mechanisms, some of which are still not fully understood. This includes the resolution of harmful local factors and neuroplasticity. The neural reorganization is the most critical driver of functional recovery post-stroke (16–19). However, a clearer understanding of the mechanisms underlying cerebral reorganization is required to develop more effective strategies to enhance the human brain's response to injury. The degree to which mechanisms underlying cerebral reorganization are successful is likely to depend on the functional state of the brain, the chronicity of the stroke, and the degree of damage.

The principal aim of this systematic review is to evaluate the existing literature on alterations in SMN in stroke patients, with a specific emphasis on delineating the repercussions of stroke on sensorimotor integration and its consequences on daily functioning throughout the different stages. Functional magnetic resonance imaging (fMRI) plays a crucial role in this evaluation, as it provides detailed insights into the brain's activity and connectivity patterns. fMRI studies have demonstrated that stroke can lead to significant changes in the SMN, affecting both structural and functional connectivity. These alterations can manifest as impaired sensorimotor integration, which can hinder the patient's ability to perform daily tasks effectively. By analyzing fMRI data across different stages of stroke recovery, researchers can better understand the dynamic changes in SMN and their impact on motor and sensory functions, thereby informing rehabilitation strategies to improve patient outcomes.

## 2 Methodology

### 2.1 Search strategy and study selection

This systematic review was conducted in compliance with Preferred Reporting Items for Systematic Reviews and Meta-Analyses (PRISMA) (20) and previous studies (21–24) are used as the reporting guidelines.

The National Center for Biotechnology Information (PubMed) and Scopus electronic databases were searched on April 2024, using the following keywords: “sensorimotor” or “motor cortex” or “supplementary motor area” or “sensorimotor network” and “stroke” or “acute stroke” or “ischemic stroke” or “hemorrhage stroke” and “functional MRI” or “fMRI”. Advanced search keyword chains in PubMed and Scopus used are in Supplementary Table S1. The references of the selected studies were cross-checked using Google Scholar. One independent reviewer (N.S.A.S) performed the systematic search and screened the title and abstract of the articles, with duplicates being removed. The full text of eligible papers was carefully read to decide whether the eligibility criteria were met. The second and third reviewers (H.A.M and N.Y) verified the findings and any possible discrepancies were solved if necessary. Associated articles in references and citations were manually checked through the Google Scholar database.

Inclusion criteria to select the studies: (a) adult patients with stroke (age >18 years old), (b) motor assessment using Fugl-Mayer Assessment (FMA) whole extremity. FMA is a widely used tool for assessing motor function in patients with stroke. The average FMA score is calculated based on the total score obtained from the assessment, which ranges from 0 to 226, with higher scores indicating better motor function (89). The FMA consists of five domains: motor function, sensory function, balance, range of motion, and joint pain. The FMA is a crucial tool in stroke studies due to its comprehensive, reliable, and standardized approach to evaluating motor function and other critical domains affected by stroke. Its ability to track changes over time, predict outcomes, and compare the effectiveness of interventions makes it indispensable for both clinical practice and research in stroke rehabilitation. (c) assessment using functional magnetic resonance imaging (fMRI) post-stroke, (d) comparison with healthy controls (HC), (e) original studies in English reported in peer-reviewed journals. Pre-clinical studies, reviews, articles that are not in English, and articles without full text were excluded. Articles that utilized other imaging modalities such as computed tomography (CT), positron emission tomography (PET), ultrasound, and navigated transcranial magnetic stimulation (nTMS) were also excluded. The summary of the inclusion criteria is tabulated in Table 1, PICOS strategy for the selection of the study.

**Table 1:** PICOS strategy for the selection of the study.

**PICOS**	**Criteria**
Population	Adult stroke patients (age >18 years old)
Intervention	Functional magnetic resonance imaging of sensorimotor network and Fugl-Mayer Assessment
Comparison	Healthy controls
Outcome	Motor deficits measured by clinical assessment and fMRI
Study	All studies related to stroke patients assessed with fMRI except case report, case series, all types of review, letter to editor

**Figure 1:**
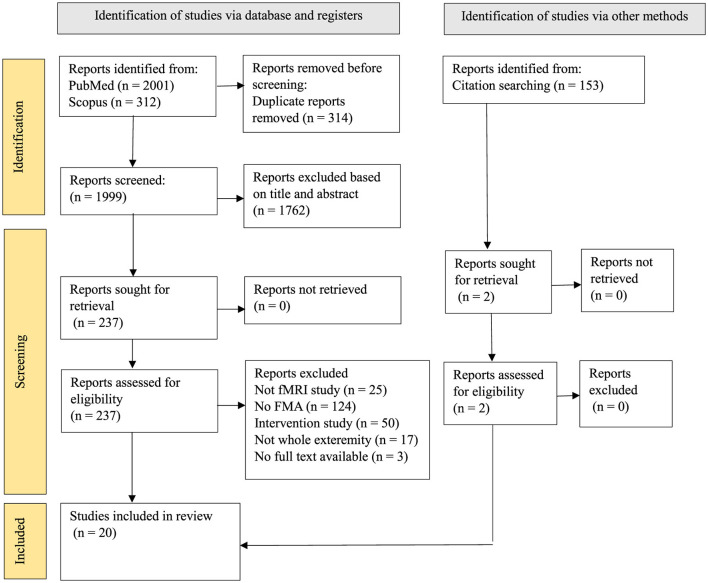
Flow diagram of the PRISMA study selection process.

The PRISMA flow diagram (Figure 1) illustrates the screening process. The electronic search identified 1999 records after removing duplicate articles, 2 other results were added through alternative sources. Thousand seven hundered and sixty-two were excluded after title and abstract screening based on the exclusion criteria. From the 237 articles selected, 219 of them were removed for not meeting the eligibility criteria, finally, 20 articles were included in this study. This systematic review was registered under the International Prospective Register of Systematic Review (PROSPERO). The tabulated data in all the tables were assessed to fulfil our primary objective. The information included (1) average FMA score at different time points and one-time points, and (2) fMRI findings after stroke onset.

### 2.2 Data extraction

After the selected articles were considered suitable for inclusion, one reviewer (N.S.A.S) extracted the following information: (a) study author(s), (b) year of publication, (c) country of origin, (d) number of participants, (e) mean age of participants ± standard deviation, (f) type of strokes, (g) post-stroke onset, (h) lesion location, (i) motor assessments, and (j) analysis of fMRI (Table 2). The second and third reviewers (H.A.M and N.Y) verified the information and any possible discrepancies were discussed and solved if necessary.

**Table 2:** Demographic of selected studies.

**No**.	**Author (year): country**	**No of participants**	**Mean age ±SD (age range)**	**Female (%)**	**Type of stroke**	**Post-stroke onset (days)**	**Study type**	**Lesion location**	**Motor assessments**	**fMRI analysis**
1	Li et al. (2022): China	28 PT, 42 HC	54.6 (30–70)	17.9	Ischemic	< 7	Cross-sectional	BG	FMA	SBA
2	Chen et al. (2018a): China	76 PT, 55 HC	NR	43.5	Ischemic, hemorrhage	7–30	Cross-sectional	CR, IC, BG, thalamus, pons, cerebellum	FMA	SBA
3	Park et al. (2011): South Korea	12 PT, 11 HC	58.4 (47–74)	58.3	Ischemic	< 14	Longitudinal (2-week, 1-month, 3-month, 6-month)	Middle cerebral artery, CR, anterior cerebral artery, striatocapsular	FMA	NR
4	Liu et al. (2015): China	22 PT, 22 HC	NR (34–75)	36.4	Ischemic	< 7	Longitudinal (1-week, 1-month, 3-month)	Left hemisphere	FMA	ALFF, cortical thickness
5	Chen et al. (2019): China	31 PT, 20 HC	NR (40–80)	32.3	Ischemic	>30	Cross-sectional	BG, pontine	FMA	SBA
6	Cheng et al. (2015): China	12 PT, 16 HC	61.5 (47–77)	33.3	Ischemic	< 90	Longitudinal (10-day. 2-week, 1-month, and 3-month)	BG, parietal lobe, CR, lateral ventricle	FMA	ROI-based
7	Wang et al. (2022): China	47 PT, 56 HC	57.8 (40–80)	44.7	Ischemic	>180	Cross-sectional	Pontine	FMA, FT, NBT, SBT, TMT	ICA, intra- and inter-network
8	Lee et al. (2018): South Korea	40 PT, 24 HC	NR (33–79)	37.5	Ischemic	>14	Longitudinal (2-weeks, 3-months)	Supratentorial, infratentorial	FMA	ROI-based
9	Lu et al. (2019): China	17 PT, 17 HC	52.0 (18–65)	5.8	Ischemic	7–90	Longitudinal (1-week, 1-month, 3-month)	IC	FMA	ICA, ROI-based, intra-network
10	Diao et al. (2020): China	86 PT, 75	NR (40–75)	25.6	Ischemic	>180	Cross-sectional	IC, BG, thalamus	FMA, TMT	SBA
11	Wang et al. (2014): China	25 PT, 22 HC	56.2 (42–72)	28.0	Ischemic	>180	Cross-sectional	IC, CR, BG, thalamus	FMA	ICA, intra- and inter-network
12	Miyai et al. (2001): Japan	18 PT, 5 HC	NR (38–71)	38.9	Ischemic	< 140	Cross-sectional	IC	FMA	ROI-based
13	Liu et al. (2020): China	25 PT, 22 HC	56.2 (42–72)	28.0	Ischemic	>180	Cross-sectional	IC, CR LN, thalamus, Cau	FMA	ROI-based
14	Li et al. (2020): China	25 PT, 26 HC	52.7 (NR)	28.0	Ischemic	< 180	Longitudinal (7-day, 2-week, 1-month, 3-month, 6-month)	BG	FMA, grip strength	Voxel-based, LI
15	Chen et al. (2023): China	34 PT, 44 HC	56.5 (30–75)	26.4	Ischemic	< 10	Longitudinal (2-week, 3-month, 6-month)	BG, CR	FMA	DC
16	Wei et al. (2020): China	20 PT, 20 HC	NR (40–80)	45.0	Ischemic	< 7	Longitudinal (1-week, 1-month, 3-month, 6-month)	Pontine	FMA	SBA
17	Kalinosky et al. (2019): USA	10 PT, 21 HC	66.7 (NR)	40.0	Ischemic	< 180	Cross-sectional	Temporal lobe, pons, IC, CST, pre-CG, post-CG	FMA, Wolf motor, box and blocks	ICA
18	Chen et al. (2018b): China	42 PT, 55 HC	57.9 (40–80)	50.0	Ischemic, hemorrhage	< 14	Cross-sectional	CR, IC, BG thalamus	FMA	SWB
19	Hong et al. (2022): China	36 PT, 38 HC	56.7 (NR)	8.3	Ischemic	>90	Cross-sectional	Sub-cortical	FMA	ROI-based

PT, patient; HC, healthy controls; SD, standard deviation; NR, not recorded; BG, basal ganglia; CR, corona radiata; IC, internal capsule; CG, central gyrus; FMA, Fugl-Mayer Assessment; FT, Flanker Task; NBT, Number back Task; SBT, Spatial back Task; TMT, Trail Making Test; VBM, voxel-based morphometry; GLM, general linear model; SBA, seed-based analysis; ROI, region of interest; ICA, independent component analysis; DC, degree centrality; SWB, sliding-window based analysis.

### 2.3 Quality assessment

Assessment tools from the National Heart, Lung and Blood Institute—Quality Assessment Tool for Observational Cohort and Cross-Sectional Studies—have been used to assess the quality of included studies. Details of the study selection assessment can be found in the Supplementary Table S2.

## 3 Results

A total of 20 studies that match the PICOS criteria were selected in this study. Tables 3, 4 summarize the study's characteristics; investigating the alterations of SMN due to stroke. The selected studies comprise 1,224 participants: 618 patients with different types and locations of stroke, and 606 healthy controls (HC). Generally, all the selected studies have moderate and high quality, as shown in Supplementary Table S2. The sample data collection techniques, and study designs used in the selected studies adhere to the established standards. All studies involved adult patients with stroke in various locations and used reliable and valid data collection methods.

**Table 3:** Motor assessments and fMRI findings in different time points.

**Author(s)**	**Average FMA score (range)**				**fMRI findings**

	**1–2 weeks post-stroke**	**1-month post-stroke**	**3-months post-stroke**	**6-months post-stroke**	
Park et al. (2011): South Korea	24.2 (8–52)	30.8 (8–59)	50.5 (17–100)	53.8 (21–100)	Increased connectivity of ipsilesional M1 with cerebellum, thalamus and MFG since onset
Cheng et al. (2015): China	72.8 (50–95)	77.1 (53–95)	81.4 (57–99)	NR	Several functional connections strongly correlated with recovery time
Lee et al. (2018): South Korea	STS: 44.5 (11–83) ITS: 44.8 (12–75)	NR	STS: 66.8 (24–100) ITS: 71.7 (26–100)	NR	Network distance and interhemispheric connectivity were significantly disrupted in STS group after stroke onset
Lu et al. (2019): China	72.4 (NR)	81.0 (NR)	95.0 (NR)	NR	Altered intra-network observed in PT compared to HC
Li et al. (2020): China	64.3 (NR)	68.4 (NR)	69.2 (NR)	73.2 (NR)	PT exhibit increased FC in MPFC and MFG
Wei et al. (2020): China	LPI: 73.9 (NR) RPI: 83.2 (NR)	LPI: 85.9 (NR) RPI: 96.3 (NR)	LPI: 89.6 (NR) RPI: 98.4 (NR)	LPI: 91.2 (NR) RPI: 99.0 (NR)	Altered CBF and FC found in PT

FMA, Fugl-Mayer Assessment; fMRI, functional magnetic resonance imaging; NR, not recorded; PT, patients; STS, supratentorial stroke; ITS, infratentorial stroke; LPI, left pontine infarction; RPI, right pontine infarction; HC, healthy controls; MFG, middle frontal gyrus; FC, functional connectivity; MPFC, middle prefrontal cortex; CBF, cerebral blood flow.

**Table 4:** Motor assessments and fMRI findings in one-time point.

**Author(s)**	**Average FMA scores (range)**	**fMRI findings**
Li et al. (2022): China	77.03 (NR)	PT had frequency-specific alterations in FC
Chen et al. (2018): China	Right-sided PT: 76.53 (NR) Left-sided PT: 72.24 (NR)	Patterns of both static FC and dynamic FC changed after stroke
Chen et al. (2019a): China	Pontine: 89.14 (NR) BG: 91.00 (NR)	Different patterns of FC damage observed in PT
Wang et al. (2022): China	94.89 (NR)	Altered inter-network and intra-network observed in PT
Diao et al. (2020): China	Right-sided PT: 87.1 (28–100) Left-sided PT: 92.9 (19–100)	PT with left-sided lesion exhibit stronger global- and long-range FC in SMC
Wang et al. (2014): China	98.8 (94–100)	Altered inter-network and intra-network observed in PT
Liu et al. (2020): China	98.8 (94–100)	SMA subregions and preSMA showed decreased rsFC
Chen et al. (2023): China	74.9 (NR)	Altered dynamic centrality observed in PT in comparison to HC
Chen et al. (2019b): China	76.4 (NR)	Pattern of intrinsic brain activity is altered in PT compared to HC

FMA, Fugl-Mayer Assessment; fMRI, functional magnetic resonance imaging; NR, not recorded; PT, patients; BG, basal ganglia; FC, functional connectivity; SMC, sensorimotor cortex; SMA, supplementary motor area; rsFC, resting-state functional connectivity; HC, healthy controls.

### 3.1 Study details

Table 2 provides a summary of the study characteristics and demographic data. Most of the studies were conducted in China (*n* = 16), while other studies were conducted in South Korea (*n* = 2), Japan (*n* = 1), and the United States of America (*n* = 1). Participants included in the studies have an age range between 18 and 80 years old. Most studies matched the age and gender of the HC. Additionally, none of the studies conducted separate analyses based on age and gender.

Most studies reported ischaemic stroke, while only two studies included patients with hemorrhagic stroke (25, 26). Lesion locations were reported in multiple brain areas with basal ganglia being the most common location reported (*n* = 9), followed by internal capsule (*n* = 8) and, corona radiata (*n* = 6). Other locations include the temporal lobe, pontine, thalamus, and parietal lobe.

The research included nine longitudinal studies with follow-up periods ranging from 10-days post-stroke to 6 months (27–34).

Four cross-sectional studies recruited patients with < 2-week post-stroke onset (25, 26, 33, 35), two studies with patients within 3 months post-stroke onset (36, 37), and the remainder reported patients up until after 6 months post-stroke onset (38–43).

Seven studies provided reports on multiple scanning and motor assessment at different time points, Four studies featured other motor assessments aside from FMA, which include Wolf motor, box and blocks, grip strength, Trail Making Test, Flanker Task, Number back Task, and Spatial back Task (32, 40, 42, 43), Additionally, fMRI analysis used in each study includes Voxel-based Morphometry, General Linear Model, Seed-based Analysis, Independent Component Analysis, Degree Centrality, and Intra- and Inter-networks analysis.

### 3.2 Fugl-Mayer Assessment

#### 3.2.1 Within 2 weeks of stroke

**Figure 2:**
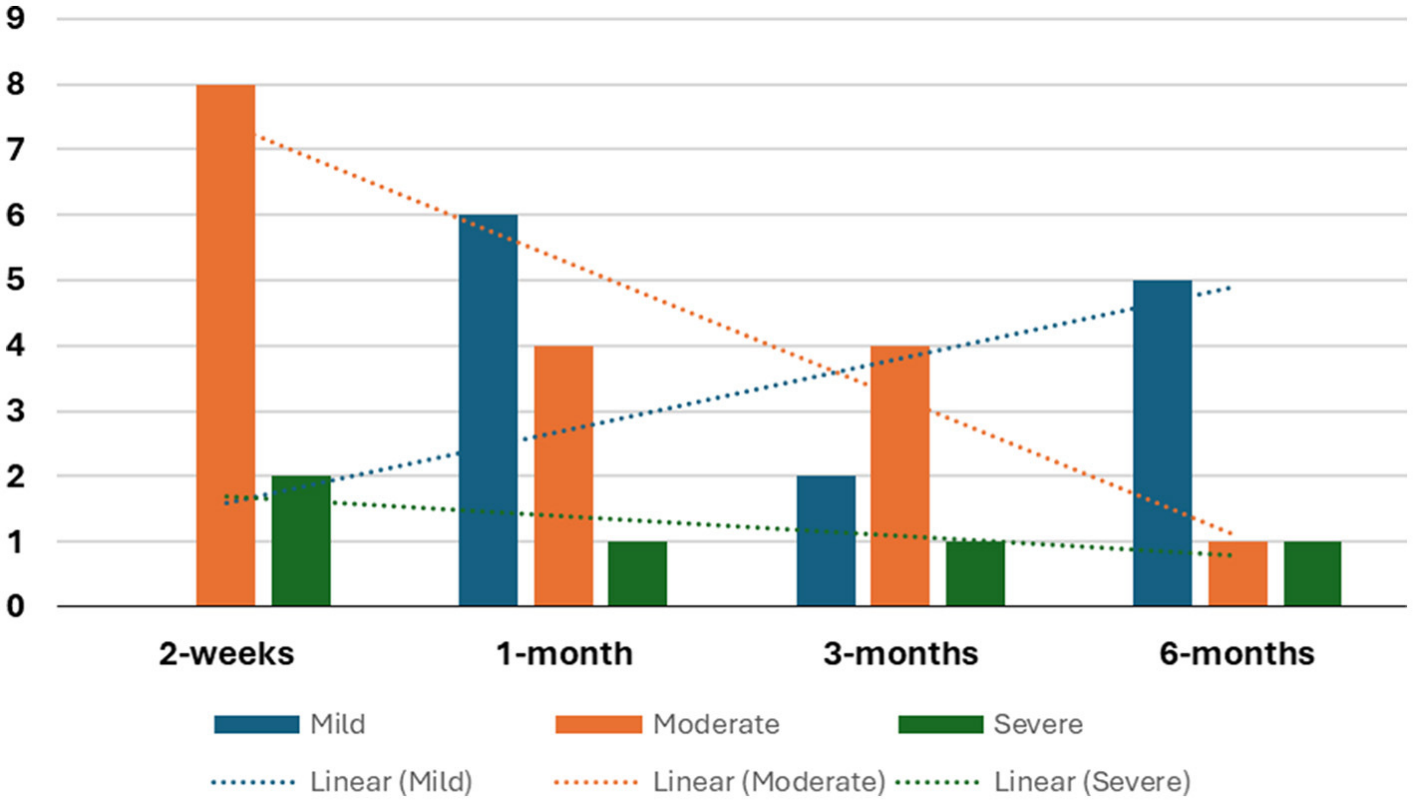
The number of studies revealing FMA scores at different time points.

Seven studies observed moderate motor impairment within 2 weeks of stroke onset (26, 28, 31–35), while two studies reported severe motor impairment at the same timeframe (27, 30). Noteworthy is Lee et al. (30), found that patients with supratentorial stroke exhibited slightly lower average FMA scores compared to patients with infratentorial stroke. The details tabulation of correlation between FMA and brain activity is in Table 5. On the other hand, Wei et al. (33) demonstrated that patients with left pontine stroke had marginally lower average FMA scores compared to their right pontine stroke counterparts. The number of studies reporting FMA scores at different time points is detailed in Figure 2.

**Table 5:** Correlation between FMA and brain activity.

**Authors**	**Stroke stages**	**Correlation fMRI findings with clinical assessment**
Chen (2018a)	Sub-acute	**Contralesional** Negative correlation between M1 and FMA
Chen (2018b)		Positive correlation between SMA and FMA
Liu (2015)	Acute to chronic	Positive correlation between SMC and FMA
Wei (2020)	Acute to chronic	Negative correlation between SMA and FMA
Li (2022)	Acute	No correlation between FC and FMA
Chen (2023)	Subacute to chronic	Positive correlation between precentral gyrus and FMA
Cheng (2015)	Acute to chronic	**Ipsilesional** Positive correlation between FC for SMN and FMA
Wu (2017)	Chronic	Negative correlation between aGMV and FMA
Lu (2019)	Acute to chronic	No correlation between SMN FC and FMA
Miyai (2001)	Chronic	
Wang (2014)	Subacute	No correlation mentions between region and FMA
Chen (2019)		
Kalinosky (2019)	Chronic	No correlation between region and FMA
Liu (2019)	Chronic	No correlation mention
Diao (2020)	Chronic	No correlation between SMN FC strength and FMA
Li (2020)	Acute to chronic	No correlation between FC strength and FMA
Wang (2022)	Chronic	
Park (2011)	Chronic	Positive correlation between SMN region and FMA
Lee (2018)	Subacute to chronic	Positive correlation between altered network and FMA

FMA, Fugl-Meyer Assessment; SMA, supplementary motor area; PMC, premotor cortex; FC, functional connectivity; SMC, supplementary motor cortex; _R, right side; _L, left side; TH, thalamus; aGMV, average gray matter volume

#### 3.2.2 1-month stroke onset

Five studies documented patients' average FMA scores, revealing significant improvement compared to scores within the initial 2 weeks post-stroke onset (27, 28, 31–33).

Furthermore, four studies documented that despite significant improvement, patients' average scores continue to reflect moderate motor impairment (28, 31, 32). Park et al. (27) reported sustained severe motor impairment within the first month after stroke onset.

Two studies showed that left-sided lesion patients have lower average FMA scores compared to right-sided lesion patients (25, 33).

#### 3.2.3 3-months stroke onset

Six studies documented a significant improvement in patients' average scores compared to earlier assessments (27, 28, 30–33). However, only two studies categorized patients as having mild motor impairment (31, 33). Notably, Wei et al. (33) observed that left pontine stroke patients still displayed consistently lower average scores than their counterparts while Chen et al. (36) reported slightly lower scores for patients with pontine stroke compared to those with basal ganglia stroke.

Four studies indicated the persistence of moderate motor impairment in patients (25, 28, 30, 32). Lee et al. (30) found that supratentorial stroke patients had lower average recovery scores than those with infratentorial strokes, with the latter showing significant improvement within the first 2 weeks. In contrast, Park et al. (27) reported lingering severe motor impairment 3 months post-stroke, although acknowledging a substantial improvement compared to the first month.

#### 3.2.4 6-months stroke onset

Among the seven studies longitudinal studies, only three studies followed up patients up to the 6-month mark post-stroke (27, 32, 33). Two studies, although reported improvement in FMA scores during this period remained in the same category, Park et al. (27) in severe group while Li et al. (32) in the moderate group. In studies employing one-time point assessments, four studies identified mild motor impairment in patients (36, 41–43).

Wei et al. (33) found near-normal motor recovery in right pontine stroke patients, while Diao et al. (42) reported slightly better outcomes for left-sided strokes.

### 3.3 Resting state-FMRI analysis

FMRI analysis used includes General Linear Model (*n* = 1), Seed-based Analysis (*n* = 10), Independent Component Analysis (*n* = 5), Degree Centrality (*n* = 1), and Intra- and Inter-networks analysis (*n* = 3).

Two studies revealed asymmetrical resting-state connectivity and altered functional connectivity (26, 27). Three studies mentioned diminished functional connectivity (FC) in multiple locations and several networks, including intra-hemispheric networks (32, 40, 43) and one study reported a decreased degree of centrality in multiple supratentorial locations in basal ganglia and corona radiata stroke (34). The decreased functional connectivity locations were found in the inferior occipital gyrus, medial prefrontal cortex, and middle frontal gyrus in the left basal ganglia stroke, and ipsilesional hemisphere in supratentorial stroke (32).

On the other hand, one study showed an increased degree of centrality (34) and six studies reported increased functional connectivity in multiple networks and different locations of strokes (28, 31, 32, 37, 38, 43). Cheng et al. (28) observed an initial in FC at the acute stage followed by a gradual decrease during the subacute phase of motor recovery. This finding was supported by Miyai et al. (38) demonstrating contralateral SMC activation in early subacute patients. Moreover, Liu et al. (29) also observed increased ALFF in bilateral PMC at 12 weeks post-stroke.

Li et al. (32) observed diminished functional connectivity (FC) strength in the calcarine and inferior occipital gyrus (IOG) regions, coupled with heightened FC in the medial prefrontal cortex (MPFC), middle frontal gyrus (MFG), and insula among patients with left basal ganglia stroke. Moreover, the FC strength pronounced lateralization between the bilateral cerebral hemispheres, favoring the contralesional hemisphere in patients. This is supported by Wang et al. (43) that identified diminished intra-network FC in primary perceptual and higher cognitive control networks, including the SMN, visual network (VIS), DMN, and salience network. Reduced inter-network FC was also observed in the primary perceptual (VIS-SMN) and higher cognitive control networks (43). Moreover, there was decreased intra-network FC in three left hemispheric brain regions (left paracentral lobule, left praecuneus, and left middle cingulum (43) and within the frontoparietal network and anterior DMN compared to HC (39). Kalinosky et al. (40) also found decreased inter-network FC between the ipsilesional SMC and the contralesional cerebellum which notably correlated with task performance, specifically hand function scores in the box and blocks scores. Subsequently, Park et al. (27) also showed decreased connectivity with the SMC, occipital cortex, and MFG could persist over 6 months after stroke.

On the other hand, Lu et al. (31) reported heightened intra-network FC in two right hemispheric regions (right PreCG and right paracentral lobule) among patients compared to HC. Wang et al. (39) also found that stroke patients had increased intra-network FC in the SMN, VIS, auditory network, dorsal attention network, and DMN. This finding is supported by Cheng et al. (28) who observed an initial increase in FC at the acute stage, followed by a gradual decrease during the sub-acute phase of motor recovery, and Miyai et al. (38) who demonstrated contralateral SMC activation in early subacute patients, accompanied by frequent ipsilateral SMC activation. Two other studies also supported the findings: Hong et al. (37) identified specific FC increases between the cerebellar anterior lobe and cerebellar posterior lobe with cerebral regions in chronic stroke patients compared to HC, and Liu et al. (29) showed an increased amplitude of low-frequency fluctuations (ALFF) was observed only in bilateral M1 and only at 12 weeks post-stroke, as also reported by Chen et al. (26) about significant altered dynamic ALFF in various brain regions in patients compared to HC. Then, Park et al. (27) also showed a persisting findings for increased connectivity in the cerebellum, thalamus, and posterior parietal cortex over 6 months after stroke.

Liu et al. (41) reported distinct functional reorganization patterns within the SMA, with the SMA proper exhibiting increased resting-state FC (rs-FC) with the primary sensorimotor area and caudal cingulate motor area, while the preSMA showed increased rs-FC with the rostral cingulate motor area of the motor control network. Moreover, both SMA subregions displayed decreased rs-FC with the fronto-insular cortex (41). Then, Chen et al. (34) noted an increased degree of centrality in the right superior temporal gyrus and the left praecuneus, while decreases were observed in the right inferior temporal gyrus, right IOG, right PreCG, and right SMA.

### 3.4 Correlation analysis

Several studies performed correlation analysis between neurophysiological assessment and fMRI findings, particularly emphasizing the precentral gyrus (PreCG) and supplementary motor area (SMA) as key components of the SMN. Notably, conflicting reports exist regarding its relationship with the FMA scores: one study found a negative correlation with the contralesional PreCG (25), while another study reported a positive correlation (34). Additionally, two studies reported positive correlations between SMC-M1 connectivity with the FMA scores (27, 29). The SMA also demonstrated mixed results: one study reported a positive correlation (26), while another found a negative correlation (33). These discrepancies suggest a complex and context-dependent role for the PreCG and SMA in sensorimotor functions and recovery.

Furthermore, seven studies reported no correlation between the SMN and the FMA scores (31, 32, 35, 39, 40, 42) indicating variability across different populations or methodologies.

Moreover, additional studies have linked fMRI findings with other neurophysiological measures. For instance, Wang et al. (43) found a negative correlation between FC and attention, working memory, and overall memory scores. Lee et al. (30) identified a positive correlation between motor function and altered network measures in both supratentorial and infratentorial strokes, underscoring the importance of network dynamics in understanding motor recovery.

### 3.5 Lesion location analysis

#### 3.5.1 Basal ganglia lesion

The studies by Li et al. (32), Li et al. (35), and Chen et al. (36) all investigate functional connectivity (FC) changes in patients with basal ganglia strokes, finding that the frontal cortex is particularly affected. Chen et al. (36) reported decreased FC in specific brain regions, including the left precuneus, right SMA, and right SFG. Li et al. (35) focused on frequency-specific FC changes, finding that different frequency bands (conventional, Slow-4, Slow-5) exhibited distinct patterns in FC between motor and visual regions. They consistently observed higher FC between bilateral M1 and bilateral medial SFG and lower FC with bilateral lingual gyrus across multiple frequency bands, compared to healthy controls. They also employed SVM analysis, achieving high accuracy in predicting stroke patients using combined features from the Slow-4 and Slow-5 bands. In contrast, Li et al. (32) explored longitudinal changes in FC, showing that stroke patients exhibited stronger FC in the contralesional middle prefrontal cortex (MPFC) over 6 months post-stroke, with initially stronger FC in the middle frontal gyrus (MFG) shifting to the insula at 6 months.

#### 3.5.2 Pontine lesion

Wei et al. (33), Chen et al. (36), and Wang et al. (43) all examine FC alterations in pontine stroke patients, consistently reporting decreased FC in key brain networks, particularly in language and visual networks. Both Chen et al. (36) and Wang et al. (43) showed reduced FC in the DMN. Wang et al. (43) further identified decreased intra-network FC in the DMN, VIS, and SAN networks. as well as decreased FC between perceptual and motor networks (VIS-SMN) and increased FC between perceptual and cognitive control networks (VIS-DMN, VIS-frontoparietal) were found.

A study by Wei et al. (33) uniquely explored differences in cerebral blood flow (CBF) and FC changes between left and right pontine stroke patients. In left pontine stroke patients, changes during the follow-up period were observed in regions associated with language and visual networks, specifically the contralesional supramarginal gyrus (SpMG) showed decreased CBF with time. These patients also demonstrated a recovery pattern where FC between the contralesional SpMG and the contralesional middle temporal gyrus (MTG), middle occipital gyrus (MOG), and inferior frontal gyrus (IFG) decreased until 3 months post-stroke before increasing. In contrast, right pontine stroke patients exhibited CBF changes in integrative brain regions, including increased CBF in the contralesional cingulate gyrus and middle occipital gyrus over time, while CBF in the ipsilesional SMA was initially high, decreased at 1 month, and then increased again.

#### 3.5.3 Supratentorial vs. infratentorial lesion

Lee et al. (30) found that patients with supratentorial strokes showed disrupted interhemispheric balance, with increased network distance and reduced interhemispheric connectivity strength compared to healthy controls. Infratentorial stroke patients, by contrast, exhibited minimal disruption in these measures. During recovery, only the infratentorial stroke group showed improvements, with decreased network distance and increased interhemispheric connectivity.

#### 3.5.4 Left vs. right lesion

Three studies, along with the results from Wei et al. (33) described in the pontine section, examined the effects of left- vs. right-sided lesions. Diao et al. (42) found that patients with left-sided strokes exhibited a significant increase in FC strength and long-range FC strength within the ipsilesional SMC. Additionally, left-sided stroke patients showed increased global-range FC between the ipsilesional SMC and contralesional MFG, whereas right-sided patients did not exhibit significant differences compared to healthy controls in any analysis.

A study by Chen et al. (25) found no difference between left- and right-sided stroke patients in terms of static FC increases in sensorimotor network (SMN) regions. However, when dynamic FC was analyzed, right-sided stroke patients exhibited significant increases in FC between SMN and task-positive regions, particularly the ipsilesional MOG and contralesional PreCG or MFG. Left-sided lesions, in contrast, showed increased FC between SMN and DMN regions, specifically the ipsilesional precuneus and calcarine gyrus. The increased dynamic FC was suggested to be due to rewiring in the perilesional zone.

## 4 Discussion

The studies reviewed report significant alterations in functional connectivity (FC) in various brain regions following a stroke, with notable lateralization differences between hemispheres (32). Post-stroke, dynamic FC changes are linked to task performance during recovery, highlighting the importance of connections both within and between the SMN and other networks. Distinct patterns emerge based on lesion location, where basal ganglia lesions affect SMN connections with frontal areas, and pontine lesions impact connections with the visual and language networks. Additionally, supratentorial strokes lead to more significant SMN disruptions than infratentorial strokes, and left-hemisphere lesions disrupt SMN connectivity more than right-hemisphere lesions.

### 4.1 Motor deficits related to FC changes in sensorimotor networks

Both the initial stroke lesion and subsequent changes in FC contribute significantly to motor impairments. Stroke disrupts local neural circuits and broader connectivity across brain networks, diminishing the brain's capacity to coordinate motor functions. Alterations in FC within the SMN are common post-stroke and lead to symptoms like muscle weakness, poor coordination, and abnormal muscle tone (32, 44). These changes in FC fluctuate over time and are linked to motor performance improvements, reflecting the brain's dynamic recovery processes (28, 40).

Core regions within the SMN, such as the M1, are heavily impacted by stroke, leading to motor impairments (45, 46). Additionally, reduced FC between the DLPFC and SMN has been linked to improved processing speed and decreased reliance on cognitive-motor functions in chronic stroke survivors (47). Rehabilitation targeting both motor and cognitive domains is essential for addressing the complex effects of these SMN adaptations on motor recovery (90).

### 4.2 Regions important for motor function recovery

The SMA and M1 (also referred to as PreCG) have been consistently reported to correlate significantly with FMA scores, emphasizing their role in motor recovery. Several studies also found that improvement in functional outcomes is often linked to increased activation and connection with M1 and SMA, providing a valuable marker for evaluating therapeutic interventions (48–50). These highlights the need of therapeutic strategies that engage these regions to promote neuroplasticity (51). For instance, physical therapy exercises targeting M1 can enhance strength and coordination (52, 53), while interventions focused on SMA aid in retraining movement sequencing and planning (54).

However, challenges persist, particularly in cases of unilateral damage where contralesional M1 activation may increase, potentially complicating recovery (25, 28). Notably, early changes in FC within contralesional motor networks can significantly influence recovery in severe cases, with interhemispheric network adjustments playing a key role in motor function restoration (55, 56).

The interplay between the SMN and other resting-state networks in the brain is complex, with alterations in one network exerting influence on other networks due to their interconnected nature (7, 57, 58). Beyond the SMN, stroke also affects connections between the SMN and other networks, with recovery potentially linked to restoring these inter-network connections. Wang et al. (43) and Javaheripour et al. (59) reported disrupted FC both within the SMN and between other networks post-stroke, which is associated with motor deficits and broader cognitive effects. In cases where the motor network is significantly damaged, cognitive-related networks play a vital role in supporting motor recovery (58). Additionally, research found that motor rehabilitation enhances synergy among motor, default mode, and executive control networks, further promoting recovery and emphasizing the importance of network collaboration (60). Furthermore, stronger baseline FC and more efficient network reconfigurations have been correlated with improved long-term motor recovery in stroke patients (61).

### 4.3 Comparison of stroke location

The impact of stroke on sensorimotor integration, the process of incorporating sensory inputs to shape motor output (62–64), is contingent on the lesion's location. Evident in this review when fMRI findings are separated according to the lesion location.

This review found that basal ganglia lesions prominently affect frontal regions, aligning with reports that symptoms in patients with basal ganglia lesions are associated with disruptions in non-motor frontal subcortical circuits (65). Several studies found that basal ganglia dysfunction can interfere with motor planning and execution by disrupting normal frontal lobe functions (66), as well as contribute to imbalances between facilitatory and inhibitory processes in the frontal cortex (67). This disruption can lead to deficiencies in higher-order motor control and affect the emotional and motivational components of movement (68, 69).

Pontine stroke patients on the other hand seemed to affect the visual and language network connecitivity with DMN to some extrent. Potentially due to pontine is in pathways that connect cortical areas to the cerebellum (the pontocerebellar fibers), which is essential for integrating sensory input between the cerebellum and contralateral cortical areas; lesions here lead to reduced coordination in motor and sensory processing, impacting language functions and eye movement control (70). Alteration in the DMN indicated that pontine damage is also associated with higher cognitive processes, potentially affecting attention, language processing, and sensory integration (36). Additionally, the severity of cognitive decline due to pontine infarcts has been linked to changes in SMA activation, highlighting the phenomenon of diaschisis, where remote brain areas—particularly cortical and cerebellar regions—are affected by the injury (71).

Furthermore, alterations in intra- and inter-hemispheric sensorimotor coupling post-stroke significantly influence the planning and execution of voluntary movements (72). Notably, a study by Lee et al. (30) highlighted lower interhemispheric connectivity strength in patients with supratentorial stroke compared to those with infratentorial stroke. Recovery dynamics differed, with infratentorial stroke patients demonstrating improved interhemispheric connectivity over time, contrasting the static nature observed in supratentorial stroke cases (30). Lesions in supratentorial structure have been associated with poorer functional outcomes and persistent cognitive dysfunction (10, 73).

### 4.4 Recovery mechanisms after stroke

Following a stroke, there is a dynamic evolution in FC within the brain, characterized by distinct phases. In the acute stage, immediate neural impacts disrupt FC, leading to motor impairments (1). The insult triggers excitotoxicity, causing neuronal death in affected regions (74). An ensuing inflammatory reaction exacerbates damage, disrupting normal neural network functioning and causing loss of FC in other brain regions (75, 76).

During the sub-acute phase, characterized by recovery and repair, the inflammatory response diminishes, providing a more stable environment for neural tissue repair (77). Neuroplasticity becomes more noticeable as neurons in nearby regions undergo both structural and functional modifications to make up for the functions that have been lost (78). This results in the creation or fortification of connections, which is evident in the changes in FC patterns and enhancements in motor abilities, language, or cognitive functions (78).

In the initial 1–2 weeks post-stroke, patients exhibit severe to moderate motor impairment based on the FMA score (27, 28, 30–33, 35). Subsequently, during the sub-acute phase, significant improvement occurs, influenced by factors such as lesion size, location, individual variability, and the efficacy of rehabilitation interventions. A study reported restored connectivity between contralateral regions in the language network during the sub-acute stage (79). Changes in FC, particularly in the contralesional hemisphere, are proposed compensatory mechanisms for motor impairment (55, 56). Temporal variability analysis revealed increased dynamic FC, notably in the perilesional zone, indicating compensatory reorganization and integration of rs-FC (25, 80). These findings underscore the temporal dynamics of FC alterations post-stroke, emphasizing the potential for enhance neural plasticity during early rehabilitation (30, 81–83).

In stroke recovery, the early sub-acute phase occurs from 7 days to 3 months post-stroke, characterized by significant spontaneous neurological recovery and high neuroplasticity. Whereas, the late sub-acute phase spans from 3 to 6 months post-stroke, where neurological recovery stabilizes, and the rate of improvement slows. In the late sub-acute phase post-stroke, the cerebral adaptive processes persist, albeit at a potentially decelerated rate compared to the earlier sub-acute period (19). Behavioral assessment using FMA indicates continued patient improvement, though there were no significant changes in average scores between the late and early sub-acute phases (27, 28, 30–33). This stabilization in FMA scores during the late sub-acute phase may be attributed to the gradual attenuation of the initial rapid improvements observed in the preceding sub-acute period. Compensatory changes in FC, instigated during the sub-acute phase, may persists, or undergo further refinement during the late sub-acute phase. The brain relies on these modified connectivity patterns, particularly in regions proximal to the stroke-affected area (84).

In the chronic phase of stroke, the cerebral landscape undergoes substantial adaptive transformations, culminating in a more stabilized state of recovery (85). During this period, FC achieves a state of stability, indicative of a settled pattern within the neural network organization (85). Although the tempo of neuroplasticity tends to decelerate compared to earlier phases, the brain retains a discernible degree of plasticity during the chronic phase (86). Late recovery may manifest in some individuals during the chronic phase, attributed to multifaceted factors such as sustained rehabilitation endeavors, lifestyle adjustments, or spontaneous improvements in neural functionality. A study by Park et al. (27) observed that patients in this phase exhibited moderate motor impairment, yet alterations in connectivity endured for up to 6 months post-stroke onset. Additionally, asymmetry in FC demonstrated a dynamic shift, increasing until 1 month after the stroke, followed by a subsequent decrease, implying an evolving connectivity pattern during the initial stages of recovery (27). Miyai et al. (38) contributed insights by noting that activation patterns in contralateral and ipsilateral SMC and PMC may signify variances in cortical reorganization during this chronic phase. Notably, patients with Wallerian degeneration despite achieving similar functional outcomes might necessitate more substantial cortical reorganization for motor recovery (38).

Moreover, within this phase, a considerable number of patients attain noteworthy recovery; nevertheless, some may endure enduring deficits attributed to the initial damage extent or individual variabilities in recovery capacity (64). It is also worth noting that compensatory movement patterns have the potential to impede or obstruct genuine recovery, fostering the formation of maladaptive motor programs (87, 88). Such maladaptive programs may contribute to a deficiency in limb-girdle mobility, consequently exerting a detrimental influence on the comprehensive recovery trajectory (87, 88).

### 4.5 Limitations and challenges

While this study provides valuable insights into the relationship between SMN alterations and motor recovery in stroke patients, several limitations must be acknowledged to provide a balanced interpretation of the findings.

A significant limitation is the geographical and ethnic bias in the literature reviewed. The majority of studies were conducted in Asian populations, raising concerns about the generalizability of the findings to other ethnic groups. Differences in genetic predisposition, stroke risk factors, rehabilitation accessibility, and cultural influences on recovery behavior may lead to variations in FC patterns and recovery trajectories. Future research should incorporate multi-center, cross-cultural studies to enhance external validity.

Secondly, stroke is inherently heterogeneous, with considerable variability in lesion size, location, and severity, which complicates direct comparisons across studies. Lesions affecting different regions—such as the motor cortex, basal ganglia, or pontine areas—may lead to distinct recovery patterns due to differences in neuroplastic potential. While our review attempts to account for these variations, a more systematic classification of lesion-specific FC alterations is necessary to refine predictive models for motor recovery. Additionally, although our review focuses on ischemic stroke, differences between ischemic and hemorrhagic stroke could still introduce inconsistencies, as underlying pathophysiological mechanisms and recovery trajectories differ. Future research should explore whether shared or distinct neuroplasticity mechanisms govern motor recovery in different stroke subtypes.

Thirdly, a major challenge in synthesizing findings across studies is methodological heterogeneity, including variations in imaging protocols, FC analysis techniques, sample sizes, and statistical approaches. Differences in MRI acquisition parameters, preprocessing pipelines, and functional connectivity metrics (e.g., seed-based vs. independent component analysis) can lead to variability in reported outcomes, making direct comparisons difficult. Furthermore, small sample sizes in some studies may limit statistical power and increase the risk of false-positive findings. There is a pressing need for larger, well-controlled longitudinal studies to track FC changes over time and differentiate between transient and long-term neuroplasticity effects. Establishing standardized reporting guidelines and harmonized imaging protocols across studies could help improve reproducibility and robustness in future research.

Fourthly, despite significant progress, several fundamental questions remain unanswered. The precise temporal dynamics of FC changes during different phases of stroke recovery are not fully understood. While some studies suggest early disruptions in SMN connectivity followed by later-stage compensation, the critical time windows for targeted interventions remain unclear. Additionally, the interaction between motor and cognitive networks in stroke recovery is an area that warrants deeper exploration, particularly in patients with co-occurring cognitive impairment or aphasia. Moreover, the potential clinical translation of FC findings remains a major challenge. While FC alterations provide valuable insights into brain plasticity, their direct applicability in guiding personalized rehabilitation protocols is still evolving. AI-driven predictive models, multimodal neuroimaging integration (e.g., DTI + rs-fMRI), and neuromodulation techniques (e.g., TMS, tDCS) should be further investigated to bridge the gap between theoretical findings and practical therapeutic applications.

Finally, our database search was restricted to PubMed and Scopus, which, while comprehensive, may have excluded relevant studies indexed in other databases such as Web of Science or Embase. However, to mitigate this limitation, we cross-referenced Google Scholar to identify any additional relevant studies that were not captured in our primary search.

## 5 Conclusion

The findings from the selected literature underscore the complex interplay between sensorimotor network (SMN) alterations and motor deficits in stroke patients, highlighting the critical role of the stroke location in shaping sensorimotor integration and recovery trajectories. The evidence suggests that functional connectivity (FC) undergoes dynamic reorganization post-stroke, with compensatory mechanisms facilitating adaptive neuroplasticity. However, the extent and effectiveness of these compensatory changes appear to be influenced by multiple factors, including lesion site, stroke severity, and pre-existing neural reserve. These findings have significant implications for rehabilitation strategies, as a deeper understanding of SMN reorganization could enable the development of personalized, targeted interventions that optimize motor recovery. Despite these insights, challenges remain in translating these findings into clinically viable therapeutic approaches. Current limitations include the heterogeneity of patient responses, the variability in neuroimaging methodologies, and the need for longitudinal studies to track the evolution of FC changes over time.

Future research should focus on integrating multimodal imaging techniques, such as diffusion tensor imaging (DTI), task-based fMRI, and electrophysiological assessments, to provide a more comprehensive understanding of SMN plasticity and its interaction with cognitive recovery mechanisms. Additionally, the potential of AI-driven predictive models and brain-computer interface (BCI)-based rehabilitation strategies should be explored to enhance recovery outcomes. Further studies should also investigate the role of non-invasive neuromodulation techniques, such as transcranial magnetic stimulation (TMS) and transcranial direct current stimulation (tDCS), in modulating SMN connectivity and promoting neuroplasticity. By advancing our knowledge of the neurobiological underpinnings of post-stroke recovery, future research can bridge the gap between neuroscientific discoveries and clinical application, ultimately improving functional outcomes and quality of life for stroke survivors.

## Data Availability

The original contributions presented in the study are included in the article/Supplementary material, further inquiries can be directed to the corresponding author.

## References

[1] Baldassarre A Ramsey L Rengachary J Zinn K Siegel JS Metcalf NV . Dissociated functional connectivity profiles for motor and attention deficits in acute right-hemisphere stroke. Brain. (2016) 139:2024–38. 10.1093/brain/aww107 27225794 PMC4939692

[2] Li W Huang Y Li Y Chen X. Brain network evolution after stroke based on computational experiments. PLoS ONE. (2013) 8:e82845. 10.1371/journal.pone.0082845 24376592 PMC3869721

[3] Cheng B Schlemm E Schulz R Boenstrup M Messe A Hilgetag C . Altered topology of large-scale structural brain networks in chronic stroke. Brain Commun. (2019) 1:fcz020. 10.1093/braincomms/fcz020 32954263 PMC7425306

[4] Hatsopoulos NG Suminski AJ. Sensing with the motor cortex. Neuron. (2011) 72:477–87. 10.1016/j.neuron.2011.10.020 22078507 PMC3263767

[5] Landelle C Lungu O Vahdat S Kavounoudias A Marchand-Pauvert V De Leener B . Investigating the human spinal sensorimotor pathways through functional magnetic resonance imaging. Neuroimage. (2021) 245:118684. 10.1016/j.neuroimage.2021.118684 34732324

[6] Seidler RD Carson RG. Correction to: Sensorimotor learning: neurocognitive mechanisms and individual differences. J Neuroeng Rehabil. (2017) 14:100. 10.1186/s12984-017-0311-5 28969657 PMC5625831

[7] Lam TK Dawson DR Honjo K Ross B Binns MA Stuss DT . Neural coupling between contralesional motor and frontoparietal networks correlates with motor ability in individuals with chronic stroke. J Neurol Sci. (2018) 384:21–9. 10.1016/j.jns.2017.11.007 29249372

[8] Bostan AC Strick PL. The basal ganglia and the cerebellum: nodes in an integrated network. Nat Rev Neurosci. (2018) 19:338–50. 10.1038/s41583-018-0002-7 29643480 PMC6503669

[9] Woodlee MT Asseo-García AM Zhao X Liu SJ Jones TA Schallert T. Testing forelimb placing “across the midline” reveals distinct, lesion-dependent patterns of recovery in rats. Exp Neurol. (2005) 191:310–7. 10.1016/j.expneurol.2004.09.005 15649486

[10] Laredo C Zhao Y Rudilosso S Renu A Pariente JC Chamorro A . Prognostic significance of infarct size and location: the case of insular stroke. Sci Rep. (2018) 8:8949. 10.1038/s41598-018-27883-3 29934530 PMC6015086

[11] Semrau JA Herter TM Scott SH Dukelow SP. Robotic identification of kinesthetic deficits after stroke. Stroke. (2013) 44:3414–21. 10.1161/STROKEAHA.113.002058 24193800

[12] Kato H Izumiyama M. Impaired motor control due to proprioceptive sensory loss in a patient with cerebral infarction localized to the postcentral gyrus. J Rehabil Med. (2015) 47:187–90. 10.2340/16501977-1900 25268114

[13] Piscitelli D. Neurorehabilitation: bridging neurophysiology and clinical practice. Neurol Sci. (2019) 40:2209–11. 10.1007/s10072-019-03969-2 31190252

[14] Cumming TB Marshall RS Lazar RM. Stroke, cognitive deficits, and rehabilitation: still an incomplete picture. Int J Stroke. (2013) 8:38–45. 10.1111/j.1747-4949.2012.00972.x 23280268

[15] Cheng T Huang XD Hu XF Wang SQ Chen K Wei JA . Physical exercise rescues cocaine-evoked synaptic deficits in motor cortex. Mol Psychiatry. (2021) 26:6187–97. 10.1038/s41380-021-01336-2 34686765

[16] Carmichael ST. Cellular and molecular mechanisms of neural repair after stroke: making waves. Ann Neurol. (2006) 59:735–42. 10.1002/ana.20845 16634041

[17] Rehme AK Eickhoff SB Wang LE Fink GR Grefkes C. Dynamic causal modeling of cortical activity from the acute to the chronic stage after stroke. Neuroimage. (2011) 55:1147–58. 10.1016/j.neuroimage.2011.01.014 21238594 PMC8053821

[18] Ward N. Assessment of cortical reorganisation for hand function after stroke. J Physiol. (2011) 589:5625–32. 10.1113/jphysiol.2011.220939 22063630 PMC3249038

[19] Grefkes C Fink GR. Recovery from stroke: current concepts and future perspectives. Neurol Res Pract. (2020) 2:17. 10.1186/s42466-020-00060-6 33324923 PMC7650109

[20] Moher D Liberati A Tetzlaff J Altman DG Group P. Preferred reporting items for systematic reviews and meta-analyses: the PRISMA statement. BMJ. (2009) 339:b2535. 10.1136/bmj.b2535 19622551 PMC2714657

[21] Manan HA Yahya N. Ageing and olfactory dysfunction in trisomy 21: a systematic review. Brain Sci. (2021) 11*:70952*. 10.3390/brainsci11070952 34356186 PMC8305843

[22] Yahya N Manan HA. Neurocognitive impairment following proton therapy for paediatric brain tumour: a systematic review of post-therapy assessments. Support Care Cancer. (2021) 29:3035–47. 10.1007/s00520-020-05808-z 33040284

[23] Manan HA Yahya N Han P Hummel T. A systematic review of olfactory-related brain structural changes in patients with congenital or acquired anosmia. Brain Struct Funct. (2022) 227:177–202. 10.1007/s00429-021-02397-3 34635958 PMC8505224

[24] Yap KH Abdul Manan H Yahya N Azmin S Mohamed Mukari SA Mohamed Ibrahim N. Magnetic resonance imaging and its clinical correlation in spinocerebellar ataxia type 3: a systematic review. Front Neurosci. (2022) 16:859651. 10.3389/fnins.2022.859651 35757531 PMC9226753

[25] Chen J Sun D Shi Y Jin W Wang Y Xi Q . Alterations of static functional connectivity and dynamic functional connectivity in motor execution regions after stroke. Neurosci Lett. (2018) 686:112–21. 10.1016/j.neulet.2018.09.008 30195973

[26] Chen J Sun D Shi Y Jin W Wang Y Xi Q . Dynamic alterations in spontaneous neural activity in multiple brain networks in subacute stroke patients: a resting-state fMRI study. Front Neurosci. (2018) 12:994. 10.3389/fnins.2018.00994 30666181 PMC6330292

[27] Park CH Chang WH Ohn SH Kim ST Bang OY Pascual-Leone A . Longitudinal changes of resting-state functional connectivity during motor recovery after stroke. Stroke. (2011) 42:1357–62. 10.1161/STROKEAHA.110.596155 21441147 PMC3589816

[28] Cheng L Wu Z Sun J Fu Y Wang X Yang GY . Reorganization of motor execution networks during sub-acute phase after stroke. IEEE Trans Neural Syst Rehabil Eng. (2015) 23:713–23. 10.1109/TNSRE.2015.2401978 26151748

[29] Liu G Dang C Peng K Xie C Chen H Xing S . Increased spontaneous neuronal activity in structurally damaged cortex is correlated with early motor recovery in patients with subcortical infarction. Eur J Neurol. (2015) 22:1540–7. 10.1111/ene.12780 26453239

[30] Lee J Lee A Kim H Chang WH Kim YH. Differences in motor network dynamics during recovery between supra- and infra-tentorial ischemic strokes. Hum Brain Mapp. (2018) 39:4976–86. 10.1002/hbm.24338 30120859 PMC6866462

[31] Lu Q Huang G Chen L Li W Liang Z. Structural and functional reorganization following unilateral internal capsule infarction contribute to neurological function recovery. Neuroradiology. (2019) 61:1181–90. 10.1007/s00234-019-02278-x 31399852

[32] Li QG Zhao C Shan Y Yin YY Rong DD Zhang M . Dynamic neural network changes revealed by voxel-based functional connectivity strength in left basal ganglia ischemic stroke. Front Neurosci. (2020) 14:526645. 10.3389/fnins.2020.526645 33071728 PMC7533550

[33] Wei Y Wu L Wang Y Liu J Miao P Wang K . Disrupted regional cerebral blood flow and functional connectivity in pontine infarction: a longitudinal MRI study. Front Aging Neurosci. (2020) 12:577899. 10.3389/fnagi.2020.577899 33328960 PMC7710811

[34] Chen J Li J Qiao F Shi Z Lu W. Effects of home-based telerehabilitation on dynamic alterations in regional intrinsic neural activity and degree centrality in stroke patients. PeerJ. (2023) 11:e15903. 10.7717/peerj.15903 37671362 PMC10476610

[35] Li J Cheng L Chen S Zhang J Liu D Liang Z . Functional connectivity changes in multiple-frequency bands in acute basal ganglia ischemic stroke patients: a machine learning approach. Neural Plast. (2022) 2022:1560748. 10.1155/2022/1560748 35356364 PMC8958111

[36] Chen H Shi M Zhang H Zhang YD Geng W Jiang L . Different patterns of functional connectivity alterations within the default-mode network and sensorimotor network in Basal Ganglia and pontine stroke. Med Sci Monit. (2019) 25:9585–93. 10.12659/MSM.918185 31838483 PMC6929567

[37] Hong W Du Y Xu R Zhang X Liu Z Li M . Altered cerebellar functional connectivity in chronic subcortical stroke patients. Front Hum Neurosci. (2022) 16:1046378. 10.3389/fnhum.2022.1046378 36438634 PMC9691772

[38] Miyai I Suzuki T Mikami A Kubota K Volpe BT. Patients with capsular infarct and Wallerian degeneration show persistent regional premotor cortex activation on functional magnetic resonance imaging. J Stroke Cerebrovasc Dis. (2001) 10:210–6. 10.1053/jscd.2001.30731 17903826

[39] Wang C Qin W Zhang J Tian T Li Y Meng L . Altered functional organization within and between resting-state networks in chronic subcortical infarction. J Cereb Blood Flow Metab. (2014) 34:597–605. 10.1038/jcbfm.2013.238 24398939 PMC3982082

[40] Kalinosky BT Vinehout K Sotelo MR Hyngstrom AS Schmit BD. Tasked-based functional brain connectivity in multisensory control of wrist movement after stroke. Front Neurol. (2019) 10:609. 10.3389/fneur.2019.00609 31263444 PMC6585311

[41] Liu H Cai W Xu L Li W Qin W. Differential reorganization of SMA subregions after stroke: a subregional level resting-state functional connectivity study. Front Hum Neurosci. (2019) 13:468. 10.3389/fnhum.2019.00468 32184712 PMC7059000

[42] Diao Q Liu J Zhang X. Enhanced positive functional connectivity strength in left-sided chronic subcortical stroke. Brain Res. (2020) 1733:146727. 10.1016/j.brainres.2020.146727 32061738

[43] Wang Y Wang C Wei Y Miao P Liu J Wu L . Abnormal functional connectivities patterns of multidomain cognitive impairments in pontine stroke patients. Hum Brain Mapp. (2022) 43:4676–88. 10.1002/hbm.25982 35770854 PMC9491282

[44] Lariviere S Ward NS Boudrias MH. Disrupted functional network integrity and flexibility after stroke: relation to motor impairments. Neuroimage Clin. (2018) 19:883–91. 10.1016/j.nicl.2018.06.010 29946512 PMC6008503

[45] Rehme AK Fink GR von Cramon DY Grefkes C. The role of the contralesional motor cortex for motor recovery in the early days after stroke assessed with longitudinal FMRI. Cereb Cortex. (2011) 21:756–68. 10.1093/cercor/bhq140 20801897

[46] Volz LJ Rehme AK Michely J Nettekoven C Eickhoff SB Fink GR . Shaping early reorganization of neural networks promotes motor function after stroke. Cereb Cortex. (2016) 26:2882–94. 10.1093/cercor/bhw034 26980614 PMC4869817

[47] Andrushko JW Rinat S Greeley B Larssen BC Jones CB Rubino C . Improved processing speed and decreased functional connectivity in individuals with chronic stroke after paired exercise and motor training. Natureportfolio. (2023) 13:13652. 10.1038/s41598-023-40605-8 PMC10444837 37608062

[48] Askim T Indredavik B Vangberg T Haberg A. Motor network changes associated with successful motor skill relearning after acute ischemic stroke: a longitudinal functional magnetic resonance imaging study. Neurorehabil Neural Repair. (2009) 23:295–304. 10.1177/1545968308322840 18984831

[49] Hinton DC Thiel A Soucy JP Bouyer L Paquette C. Adjusting gait step-by-step: Brain activation during split-belt treadmill walking. Neuroimage. (2019) 202:116095. 10.1016/j.neuroimage.2019.116095 31430533

[50] Ismail UN Yahya N Manan HA. Investigating functional connectivity related to stroke recovery: a systematic review. Brain Res. (2024) 1840:149023. 10.1016/j.brainres.2024.149023 38815644

[51] Michels L Dietz V Schattin A Schrafl-Altermatt M. Neuroplastic changes in older adults performing cooperative hand movements. Front Hum Neurosci. (2018) 12:488. 10.3389/fnhum.2018.00488 30618675 PMC6300783

[52] Tyc F Boyadjian A. Plasticity of motor cortex induced by coordination and training. Clin Neurophysiol. (2011) 122:153–62. 10.1016/j.clinph.2010.05.022 21168091

[53] Hand BJ Opie GM Sidhu SK Semmler JG. Motor cortex plasticity and visuomotor skill learning in upper and lower limbs of endurance-trained cyclists. Eur J Appl Physiol. (2022) 122:169–84. 10.1007/s00421-021-04825-y 34618222

[54] Ding H Seusing N Nasseroleslami B Anwar AR Strauss S Lotze M . The role of ipsilateral motor network in upper limb movement. Front Physiol. (2023) 14:1199338. 10.3389/fphys.2023.1199338 37465697 PMC10351419

[55] Buetefisch CM. Role of the contralesional hemisphere in post-stroke recovery of upper extremity motor function. Front Neurol. (2015) 6:214. 10.3389/fneur.2015.00214 26528236 PMC4607877

[56] Dodd KC Nair VA Prabhakaran V. Role of the contralesional vs. ipsilesional hemisphere in stroke recovery. Front Hum Neurosci. (2017) 11:469. 10.3389/fnhum.2017.00469 28983244 PMC5613154

[57] Duncan ES Small SL. Changes in dynamic resting state network connectivity following aphasia therapy. Brain Imaging Behav. (2018) 12:1141–9. 10.1007/s11682-017-9771-2 29064020

[58] Lee J Kim YH. Does a cognitive network contribute to motor recovery after ischemic stroke? Neurorehabil Neural Repair. (2023) 37:458–65. 10.1177/15459683231177604 37269123

[59] Javaheripour N Li M Chand T Krug A Kircher T Dannlowski U . Altered resting-state functional connectome in major depressive disorder: a mega-analysis from the PsyMR consortium. Transl Psychiatry. (2021) 11:1–9. 10.1038/s41398-021-01619-w 34620830 PMC8497531

[60] Pirovano I Antonacci Y Mastropietro A Bara C Sparacino L Guanziroli E . Rehabilitation modulates high-order interactions among large-scale brain networks in subacute stroke. IEEE Trans Neural Syst Rehabil Eng. (2023) 31:4549–60. 10.1109/TNSRE.2023.3332114 37955999

[61] Cheng HJ Ng KK Qian X Ji F Lu ZK Teo WP . Task-related brain functional network reconfigurations relate to motor recovery in chronic subcortical stroke. Sci Rep. (2021) 11:8442. 10.1038/s41598-021-87789-5 33875691 PMC8055891

[62] Carey L Matyas T. Training of somatosensory discrimination after stroke facilitation of stimulus generalization. Am J Phys Med Rehabil. (2005) 84:428–42. 10.1097/01.PHM.0000159971.12096.7F 15905657

[63] Laible M Grieshammer S Seidel G Rijntjes M Weiller C Hamzei F. Association of activity changes in the primary sensory cortex with successful motor rehabilitation of the hand following stroke. Neurorehabil Neural Repair. (2012) 26:881–8. 10.1177/1545968312437939 22396499

[64] Borich M Brodies S Gray W Ionta S Boyd LA. Understanding the role of the primary somatosensory cortex: Opportunities for rehabilitation. Neuropsychologia. (2015) 79:246–55. 10.1016/j.neuropsychologia.2015.07.007 26164474 PMC4904790

[65] Ward P Seri Cavanna AE. Functional neuroanatomy and behavioural correlates of the basal ganglia: evidence from lesion studies. Behav Neurol. (2013) 26:219–23. 10.1155/2013/616741 22713407 PMC5215714

[66] Rowe J Rittman T. The basal ganglia in cognitive disorders. In:Husain M Schott JM, editors. Oxford Textbook of Cognitive Neurology and Dementia. Oxford University Press (2016).

[67] Leisman G Braun-Benjamin O Melillo R. Cognitive-motor interactions of the basal ganglia in development. Front Syst Neurosci. (2014) 8:16. 10.3389/fnsys.2014.00016 24592214 PMC3923298

[68] Groenewegen HJ. The basal ganglia and motor control. Neural Plast. (2003) 10:107–20. 10.1155/NP.2003.107 14640312 PMC2565420

[69] Adam R Leff A Sinha N Turner C Bays P Draganski B . Dopamine reverses reward insensitivity in apathy following globus pallidus lesions. Cortex. (2013) 49:1292–303. 10.1016/j.cortex.2012.04.013 22721958 PMC3639369

[70] Schmahmann JD Ko R MacMore J. The human basis pontis: motor syndromes and topographic organization. Brain. (2004) 127:1269–91. 10.1093/brain/awh138 15128614

[71] Shimmyo K Obayashi S. Fronto-cerebellar diaschisis and cognitive dysfunction after pontine stroke: a case series and systematic review. Biomedicines. (2024) 12*:30623*. 10.3390/biomedicines12030623 PMC10967718 38540236

[72] Bolognini N Russo C Edwards D. The sensory side of post-stroke motor rehabilitation. Restor Neurol Neurosci. (2016) 11:571–86. 10.3233/RNN-150606 PMC5605470 27080070

[73] Schmidt RA Lee TD. Motor Control and Learning: A Behavioral Emphasis (4th ed). Human Kinetics (2005).

[74] Coultrap SJ Vest RS Ashpole NM Hudmon A Bayer KU. CaMKII in cerebral ischemia. Acta Pharmacol Sin. (2011) 32:861–72. 10.1038/aps.2011.68 21685929 PMC3846291

[75] Akiyama N. Herpes zoster infection complicated by motor paralysis. J Dermatol. (2015) 27:252–7. 10.1111/j.1346-8138.2000.tb02160.x 10824489

[76] Kaur GD. Cognitivism or situated-distributed cognition? Assessing kashmiri carpet weaving practice from the two theoretical paradigms. Rev Philos Psychol. (2020) 11:917–37. 10.1007/s13164-019-00455-8

[77] Dokalis N Prinz M. Resolution of neuroinflammation: mechanisms and potential therapeutic option. Semin Immunopathol. (2019) 41:699–709. 10.1007/s00281-019-00764-1 31705317

[78] Lee BH Lim T-H Yoon YW Yenari MA Jeong Y. Postinjury neuroplasticity in central neural networks. Neural Plasticity. (2015) 2015:857085. 10.1155/2015/857085 26557389 PMC4628645

[79] Stockbridge MD Faria AV Fridriksson J Rorden C Bonilha L Hillis AE. Subacute aphasia recovery is associated with resting-state connectivity within and beyond the language network. Ann Clin Transl Neurol. (2023) 10:1525–32. 10.1002/acn3.51842 37403712 PMC10502663

[80] Caliandro P Vecchio F Miraglia F Reale G Marca GD La Torre G . Small-world characteristics of cortical connectivity changes in acute stroke. Neurorehabil Neural Repair. (2017) 31:81–94. 10.1177/1545968316662525 27511048

[81] Meer MPA Marel K Wang K Otte WM Bouazati S Roeling T . Recovery of sensorimotor function after experimental stroke correlates with restoration of resting-state interhemispheric functional connectivity. Compar Study. (2010) 30:3964–72. 10.1523/JNEUROSCI.5709-09.2010 20237267 PMC6632290

[82] Desowska A Turner DL. Dynamics of brain connectivity after stroke. Rev Neurosci. (2019) 26:605–23. 10.1515/revneuro-2018-0082 30768425

[83] Vicentini JE Weiler M Casseb RF Almeida S Valler L Campos BM . Subacute functional connectivity correlates with cognitive recovery six months after stroke. Neuroimage Clin. (2021) 29:102538. 10.1016/j.nicl.2020.102538 33385880 PMC7779317

[84] Olafson ER Jamison KW Sweeney EM Liu H Wang D Brus JE . Functional connectome reorganization relates to post-stroke motor recovery and structural and functional disconnection. Neuroimage. (2021) 2021:245. 10.1016/j.neuroimage.2021.118642 34637901 PMC8805675

[85] Ritzl A Meisel S Wittsack H-J Fink GR Siebler M Modder U . Development of brain infarct volume as assessed by magnetic resonance imaging (MRI): follow-up of diffusion-weighted MRI lesions. J. Magn. Reson. Imaging. (2004) 20:201–7. 10.1002/jmri.20096 15269944

[86] Gulyaeva NV Onufriev MV Moiseeva YV. Ischemic stroke, glucocorticoids, and remote hippocampal damage: a translational outlook and implications for modeling. Neurosci. (2021) 15*:781964*. 10.3389/fnins.2021.781964 34955730 PMC8695719

[87] Takeuchi N Izumi S-I. Maladaptive plasticity for motor recovery after stroke: mechanisms and approaches. Neural Plast. (2012) 2012:359728. 10.1155/2012/359728 22792492 PMC3391905

[88] Jang SH Chang CH Lee J Kim CS Seo JP Yeo SS. Functional role of the corticoreticular pathway in chronic stroke patients. Stroke. (2013) 4*:269*. 10.1161/STROKEAHA.111.000269 23444306

[89] Fugl-Meyer AR Jääskö L Leyman I Olsson S Steglind S. The post-stroke hemiplegic patient: a method for evaluation of physical performance. Scand J Rehabil Med. (1975) 7:13–31. 1135616

[90] Pippi R Giuliani U Tenore G Pietrantoni A Romeo U. What is the risk of developing medication-related osteonecrosis in patients with extraction sockets left to heal by secondary intention? A retrospective case series study. J Oral Maxillofac Surg. (2021) 79:2071–7. 10.1016/j.joms.2021.05.031 34174218

