# Supplementary material for: A systematic review of alterations in sensorimotor networks following stroke: implications for integration and functional outcomes across recovery stages
Source: Front Neurol. 2025 May 27;16:1456146. doi: 10.3389/fneur.2025.1456146 (PMC12150405; doi:10.3389/fneur.2025.1456146)
Supplement: Supplementary file 1 [file Supplementary_file_1.docx]

**SUPPLEMENTARY MATERIAL**

**Table S1: Advanced Search Keyword chain in PubMed**

| 1 | PubMed | ((((((stroke) OR (SMN)) OR (somatosensory network)) AND ((((((fmri) OR (functional mri)) OR (functional magnetic resonance imaging)) OR (fmri)) OR (functional mri)) OR (functional magnetic resonance imaging)) |
| --- | --- | --- |
| 2 | Scopus | ((((((stroke) OR (SMN)) OR (somatosensory network)) AND ((((((fmri) OR (functional mri)) OR (functional magnetic resonance imaging)) OR (fmri)) OR (functional mri)) OR (functional magnetic resonance imaging)) |

**Table S2: Quality Assessment of Selected Studies by NHLBI, NIH**

**S2.1 Quality assessment of the studies**

Y; Yes, N; No, Other (CD; cannot determine, NA; not applicable, NR; not reported)

|  | Criteria | Wu et al., 2017 | Li et al.,2022 | Chen et al., 2018 | Miyai et al., 2001 | Liu et al., 2020 | Chen et al., 2023 | Wei et al., 2020 | Kalinosky et al., 2019 |
| --- | --- | --- | --- | --- | --- | --- | --- | --- | --- |
| 1 | Was the research question or objective in this paper clearly stated and appropriate? | Y | Y | Y | Y | Y | Y | Y | Y |
| 2 | Was the study population clearly specified and defined? | Y | Y | Y | Y | Y | Y | Y | Y |
| 3 | Did the authors include a sample size justification? | N | N | N | Y | Y | Y | Y | Y |
| 4 | Were controls selected or recruited from the same or similar population that gave rise to the cases (including the same timeframe)? | Y | Y | Y | Y | Y | Y | Y | Y |
| 5 | Were the definitions, inclusion and exclusion criteria, algorithms or processes used to identify or select cases and controls valid, reliable, and implemented consistently across all study participants? | Y | Y | Y | N | N | Y | N | Y |
| 6 | Were the cases clearly defined and differentiated from controls? | Y | Y | Y | Y | Y | Y | Y | N |
| 7 | If less than 100 percent of eligible cases and/or controls were selected for the study, were the cases and/or controls randomly selected from those eligible? | N | N | N | Y | Y | Y | Y | Y |
| 8 | Was there use of concurrent controls? | N | N | N | N | N | N | N | N |
| 9 | Were the investigators able to confirm that the exposure/risk occurred prior to the development of the condition or event that defined a participant as a case? | Y | Y | Y | Y | Y | Y | Y | Y |
| 10 | Were the measures of exposure/risk clearly defined, valid, reliable, and implemented consistently (including the same time period) across all study participants? | Y | Y | Y | Y | Y | Y | Y | Y |
| 11 | Were the assessors of exposure/risk blinded to the case or control status of participants? | N | N | N | Y | N | N | Y | N |
| 12 | Were key potential confounding variables measured and adjusted statistically in the analyses? If matching was used, did the investigators account for matching during study analysis? | Y | Y | Y | Y | Y | Y | Y | Y |

**S2.1 Quality Assessment for the studies (continue)**

| No | Criteria | Park et al., 2011 | Liu et al., 2015 | Chen et al., 2019 | Cheng et al., 2015 | Wang et al., 2022 | Lee et al., 2018 | Lu et al., 2019 | Diao et al., 2020 | Wang et al., 2014 |
| --- | --- | --- | --- | --- | --- | --- | --- | --- | --- | --- |
| 1 | Was the study question or objective clearly stated? | Y | Y | Y | Y | Y | Y | Y | Y | Y |
|  | Were eligibility/selection criteria for the study population prespecified and clearly described? | Y | Y | Y | Y | Y | Y | Y | Y | Y |
| 3 | Were the participants in the study representative of those who would be eligible for the test/service/intervention in the general or clinical population of interest? | Y | Y | Y | Y | Y | Y | Y | Y | Y |
| 4 | Were all eligible participants that met the prespecified entry criteria  enrolled? | Y | Y | Y | Y | Y | Y | Y | Y | Y |
| 5 | Was the sample size sufficiently large to provide confidence in the findings? | N | N | Y | N | Y | N | Y | Y | Y |
| 6 | Was the test/service/intervention clearly described and delivered consistently across the study population? | Y | Y | Y | Y | N | Y | N | N | Y |
| 7 | Were the outcome measures prespecified, clearly defined, valid, reliable, and assessed consistently across all study participants? | Y | Y | Y | Y | Y | Y | N | Y | Y |
| 8 | Were the people assessing the outcomes blinded to the participants' exposures/interventions? | N | N | N | N | N | N | N | N | N |
| 9 | Was the loss to follow-up after baseline 20% or less? Were those lost to follow-up accounted for in the  analysis? | Y | Y | Y | Y | Y | Y | Y | Y | Y |

| 10 | Did the statistical methods examine changes in outcome measures from  before to after the intervention? Were statistical tests done that provided p values for the pre-to-post changes? | Y | Y | Y | Y | Y | Y | Y | Y | Y | Y | Y |
| --- | --- | --- | --- | --- | --- | --- | --- | --- | --- | --- | --- | --- |
| 11 | Were outcome measures of interest taken multiple times before the intervention and multiple times after the intervention (i.e., did they use an interrupted time-series design)? | N | Y | Y | Y | N | Y | N | N | Y | N | Y |
| 12 | If the intervention was conducted at a group level (e.g., a whole hospital, a community, etc.) did the statistical  analysis take into account the use of individual-level data to determine  effects at the group level? | Y | Y | Y | Y | Y | Y | Y | Y | Y | Y | Y |

Y; Yes, N; No, Other (CD; cannot determine, NA; not applicable, NR; not reported)

**S2.1 Quality Assessment for the studies (continue)**

| No | Criteria | Chen et al., 2019b | Hong et al., 2022 |
| --- | --- | --- | --- |
| 1 | Was the study question or objective clearly stated? | Y | Y |
|  | Were eligibility/selection criteria for the study population prespecified and clearly described? | Y | Y |
| 3 | Were the participants in the study representative of those who would be eligible for the test/service/intervention in the general or clinical population of interest? | Y | Y |
| 4 | Were all eligible participants that met the prespecified entry criteria  enrolled? | Y | Y |
| 5 | Was the sample size sufficiently large to provide confidence in the findings? | N | N |
| 6 | Was the test/service/intervention clearly described and delivered consistently across the study population? | Y | Y |
| 7 | Were the outcome measures prespecified, clearly defined, valid, reliable, and assessed consistently across all study participants? | Y | Y |
| 8 | Were the people assessing the outcomes blinded to the participants' exposures/interventions? | N | N |
| 9 | Was the loss to follow-up after baseline 20% or less? Were those lost to follow-up accounted for in the  analysis? | Y | Y |

| 10 | Did the statistical methods examine changes in outcome measures from  before to after the intervention? Were statistical tests done that provided p values for the pre-to-post changes? | Y | Y |
| --- | --- | --- | --- |
| 11 | Were outcome measures of interest taken multiple times before the intervention and multiple times after the intervention (i.e., did they use an interrupted time-series design)? | N | Y |
| 12 | If the intervention was conducted at a group level (e.g., a whole hospital, a community, etc.) did the statistical  analysis take into account the use of individual-level data to determine  effects at the group level? | Y | Y |

Y; Yes, N; No, Other (CD; cannot determine, NA; not applicable, NR; not reported)
